# Prevalence of Caesarean sections in Enugu, southeast Nigeria: Analysis of data from the Healthy Beginning Initiative

**DOI:** 10.1371/journal.pone.0174369

**Published:** 2017-03-29

**Authors:** Jayleen K. L. Gunn, John E. Ehiri, Elizabeth T. Jacobs, Kacey C. Ernst, Sydney Pettygrove, Katherine E. Center, Alice Osuji, Amaka G. Ogidi, Nnabundo Musei, Michael C. Obiefune, Chinenye O. Ezeanolue, Echezona E. Ezeanolue

**Affiliations:** 1 Department of Epidemiology and Biostatistics, Mel & Enid Zuckerman College of Public Health, University of Arizona, Tucson, Arizona, United States of America; 2 Department of Health Promotion Sciences, Mel & Enid Zuckerman College of Public Health, University of Arizona, Tucson, Arizona, United States of America; 3 University of Arizona Cancer Center, Tucson, Arizona, United States of America; 4 BrightOutcome, Inc., Buffalo Grove, Illinois, United States of America; 5 Prevention, Education, Treatment, Training and Research-Global Solutions-PeTR-GS, Enugu, Enugu State, Nigeria; 6 Healthy Sunrise Foundation, Castle Ridge Avenue, Las Vegas, Nevada, United States of America; 7 Institute of Human Virology, University of Maryland, Baltimore, Maryland, United States of America; 8 Department of Pediatrics, University of Nevada School of Medicine, Las Vegas, Nevada, United States of America; TNO, NETHERLANDS

## Abstract

**Background:**

In order to meet the Sustainable Development Goal to decrease maternal mortality, increased access to obstetric interventions such as Caesarean sections (CS) is of critical importance. As a result of women’s limited access to routine and emergency obstetric services in Nigeria, the country is a major contributor to the global burden of maternal mortality. In this analysis, we aim to establish rates of CS and determine socioeconomic or medical risk factors associated with having a CS in Enugu, southeast Nigeria.

**Methods:**

Data for this study originated from the Healthy Beginning Initiative study. Participant characteristics were obtained from 2300 women at baseline via a semi-structured questionnaire. Only women between the ages of 17–45 who had singleton deliveries were retained for this analysis. Post-delivery questionnaires were used to ascertain mode-of-delivery. Crude and adjusted logistic regressions with Caesarean as the main outcome are presented.

**Results:**

In this sample, 7.22% women had a CS. Compared to women who lived in an urban setting, those who lived in a rural setting had a significant reduction in the odds of having a CS (aOR: 0.58; 0.38–0.89). Significantly higher odds of having a CS were seen among those with high peripheral malaria parasitemia compared to those with low parasitemia (aOR: 1.54; 1.04–2.28).

**Conclusion:**

This study revealed that contrary to the increasing trend in use of CS in low-income countries, women in this region of Nigeria had limited access to this intervention. Increasing age and socioeconomic proxies for income and access to care (e.g., having a tertiary-level education, full-time employment, and urban residence) were shown to be key determinants of access to CS. Further research is needed to ascertain the obstetric conditions under which women in this region receive CS, and to further elucidate the role of socioeconomic factors in accessing CS.

## Background

Globally, the number of Caesarean sections (CS) has been on the rise over the last decade [1, 2, 3, 4]. While CS are potentially life-saving, the adverse maternal and perinatal outcomes when a CS is not medically necessary have become a major public health concern as the associated expenses decrease resources available for other maternal and child health interventions [5, 6]. According to the World Health Organization (WHO), as a population’s CS rates approach 10%, maternal and newborn deaths decrease [7]. A medically necessary CS can prevent maternal and infant mortality; however, there is no evidence that CS benefits women who do not require the procedure [7]. The WHO has estimated—based on rates of fistula—that in 15.5% of pregnancies in Nigeria, a CS is medically necessary [1, 8, 9]. Underutilization of CS is of particular concern in most of Africa where 7.4% of all births occurred by CS in 2014 [3].

Although most African countries have regional hospitals with surgical services available to perform CS, multiple individual and health system characteristics impede access and contribute to the delay in women seeking services during pregnancy and delivery. Thaddeus and Maine (1994) developed the “three-delay” model that has been widely accepted as a framework to explain the obstacles in obtaining adequate healthcare during pregnancy and delivery [10]. This three-tiered framework includes: delay in decisions to seek care, delays in reaching a healthcare facility, and delays in receiving adequate treatment for obstetric complications.

In sub-Saharan Africa (SSA), delayed access to healthcare services during pregnancy and delivery can be influenced by multiple factors. Lack of knowledge of the importance of perinatal care and an inability to pay for healthcare services are common reasons for delaying healthcare utilization [10]. Women also delay seeking treatment during pregnancy and delivery because of poverty, gender inequalities in household decision making, cultural barriers, and geographical and transport barriers [10]. Increased age, education and wealth are all positively associated with deciding to have a doctor present at delivery in SSA [11, 12]. When life-threatening complications occur during labor, delays in seeking adequate care can increase maternal mortality even when lifesaving CS is performed.

In 2013, Nigeria had the second highest number of maternal deaths and had the 11th highest crude birth rate, making it an important country in which to study the barriers to obtaining adequate obstetric care [13, 14]. In 2014, 2% of births in Nigeria utilized CS [3]. More than 75% of all CS in Nigeria are linked to obstetric emergencies that could have been prevented by earlier medical care [15]. Even with birth plans in place, many Nigerian women opt to deliver with an unskilled birth attendant in a setting other than a hospital because of barriers in seeking treatment, including cost and geographical/transportation difficulties [10, 15, 16]. Cultural factors such as gender inequalities and the acceptance of home deliveries, compared to hospital deliveries, also influence a woman’s decision to deliver at a healthcare facility [17]. Delays in seeking treatment results in women attempting to access care at healthcare facilities only after life-threatening complications develop. Increasing access to obstetric care, such as CS, decreases maternal and infant morbidity and mortality [18, 19]. However, fears associated with having a CS may further delay a woman’s decision to seek treatment. Common fears associated with having a CS in Nigeria include: cultural beliefs that vaginal birth is a confirmation of womanhood, stigma of being mocked by other women, death, violation of religious beliefs, post-operative pain, future infertility, expense, and medical incompetence [20–22]. A woman’s socioeconomic status also influences access to CS, with the richest women, as measured by wealth index, having better access to CS compared to the poorest women [9]. Education and age are also strong predictors of a woman’s willingness to have a CS in Nigeria [17, 18]. Women who are either younger or less educated are more likely to refuse a CS even when it is medically necessary, and this usually stems from concerns about the expense [12, 23, 24].

Even women who do attempt to access healthcare facilities during delivery, often encounter poorly trained personnel and a lack of proper equipment and supplies [12]. Most studies evaluating pregnancy outcomes in Nigeria equate having a doctor present at delivery to having access to quality healthcare. However, this metric may not be an accurate predictor of the facility’s ability to perform a CS. Many healthcare facilities within Nigeria cannot offer a CS, and ambulance services are virtually non-existent [25]. In fact, one study demonstrated that only 1 in 21 health facilities in Nigeria is equipped to perform CS [26]. This complicates the ability of pregnant women to obtain adequate healthcare during pregnancy and delivery.

In order to meet the Sustainable Development Goal target of reducing the global maternal mortality ratio to less than 70 per 100,000 live births by 2030 [27], increased in access to lifesaving obstetric measures such as CS is needed [18, 19, 28]. Examining factors associated with having a CS will help provide insight into ways to increase access to healthcare during pregnancy and delivery. In addition, socioeconomic and comorbid conditions are often not examined together when exploring factors associated with CS in Nigeria. Therefore, the aims of this paper were two-fold: 1) to establish the rates of CS in Enugu, southeast Nigeria; and 2) to determine socioeconomic or medical risk factors associated with having a CS.

## Methods

### Geographical area

Enugu State is in the southeastern part of Nigeria. With a population between 3–6 million, according the state government, Enugu has predominately rural agrarian households with some urban centers [29].

### Survey

Data for this study were derived from the Healthy Beginning Initiative study (HBI), which has been described in detail elsewhere [30]. Briefly, the parent study was a two-arm randomized cluster trial aimed to assess rates of HIV testing. HBI used congregation-based sampling to recruit pregnant women and their partners in 40 churches from four dioceses (the Anglican Diocese of Enugu, the Catholic Diocese of Enugu, the Anglican Diocese of Oji-River, and the Catholic Diocese of Agwu). Women who were self-identified as pregnant were included in the study. Recruitment occurred at the level of the churches and participants (in that order), while randomization occurred only at the church level—that is, churches were randomly assigned to either the intervention or control groups. The intervention group participated in educational games about healthy pregnancy habits in addition to HIV acquisition modes, and effective prevention of mother-to-child transmission. They were also offered free prenatal care in the form of blood samples taken on-site to test for HIV, hemoglobin, malaria, hepatitis B, sickle cell gene, and syphilis. Women who tested positive for HIV were linked to local HIV care. Women in the control group were encouraged to attend prenatal care through nearby health facilities and were also referred to the health facility for testing. The research team maintained direct contact with health facilities to confirm HIV testing and prevention of mother-to-child transmission completion. Women in both the control and intervention groups completed a post-delivery questionnaire, which was available every 2 to 3 months at church. Maternal mortality was not ascertained in the parent study.

In Nigeria, approximately 35% of pregnant women deliver at a healthcare facility; therefore, a community-based sampling technique was employed to obtain a more representative sample of pregnant women [31]. Also, because pregnant women were recruited from churches in Enugu State, Nigeria, a more representative sample of pregnant women was expected as the population is more than 95% Christian and church attendance approaches 90% [30]. Pregnant women interested in the study were asked to read and sign a consent form in either English or the local language, Ibo. If the participant was illiterate, the consent form was read aloud to her in the local language; then the participant affixed her thumb print as an indication of her consent to participate in the study [30]. Demographic characteristics of participants were obtained at baseline via a semi-structured questionnaire [30]. Trained research staff and church-based health advisors administered this questionnaire written at a 6th grade reading level. Participants had the option of reading the survey themselves or having study personnel read to them. Because of inherent risks associated with having multiples (i.e. twins, triplets etc.), only women between the ages of 17–45 at baseline, who had singleton deliveries, were retained for this analysis. The length of pregnancy at baseline was not ascertained. Participants remained in the study until post-delivery. Post-delivery questionnaires were used to ascertain the mode of delivery, i.e., CS or vaginal birth, and singleton or multiple deliveries. Gravidity was dichotomized as primigravida and multigravida. Overall 76.6% (n = 2300) of participants who gave informed consent answered questions regarding their mode of delivery (Control n = 1042; Intervention n = 1258). Only women who answered the question regarding mode of delivery on the post-delivery questionnaire were retained for the analysis described in Table 1. However, not all women had complete data on socioeconomic and comorbid conditions; therefore, only 1,680 women were retained for the analysis described in Table 2. The study took place from January 2013 to August 2014.

**Table 1:** Comparison of participant baseline characteristics and infant gender with mode of delivery.

	Full Sample					Primigravida				
	C-Section		Vaginal Birth		P*	C-Section		Vaginal Birth		P*
	N	%	N	%		N	%	N	%	
**Total**	166	7.22	2134	92.78		26	7.81	307	92.19	
**Mother's Age**										
17–24	16	3.27	473	96.73	*<0*.01*	9	5.84	145	94.16	*0*.*34*^a^
25–34	106	7.05	1308	92.50		15	9.26	147	90.74	
35–45	44	11.22	348	88.78		2	11.76	15	88.24	
**Education**										
None /Primary	30	5.03	567	94.97	*<0*.01*	3	6.00	47	94.00	*0*.*06* ^a^
Secondary	78	5.95	1234	94.05		11	5.56	187	94.44	
Tertiary	58	15.06	327	84.94		12	14.12	73	85.88	
**Employment Status**										
Full-time	74	8.72	775	91.28	*0*.03*	6	5.83	97	94.17	*0*.*63* ^a^
Part-time	28	5.01	531	94.99		6	9.52	57	90.48	
None	60	6.89	811	93.11		14	8.54	150	91.46	
**Residence**										
Urban	72	12.00	528	88.00	*<0*.01*	14	14.29	84	85.71	*<0*.01*
Rural	94	5.55	1599	94.45		12	5.11	223	94.89	
**Malaria Parasitemia**										
Low	66	6.08	1020	93.92	*0*.03*	14	8.33	154	91.67	*0*.*36*
High	58	8.80	601	91.20		5	5.26	90	94.74	
**Number of People in Household**										
1–2	30	8.55	321	91.45	*0*.*09*	14	7.49	173	92.51	*0*.*15* ^a^
3–4	75	8.20	840	91.80		11	11.58	84	88.42	
5+	60	5.91	956	94.09		1	2.13	46	97.87	
**Gravity**										
Multigravida	135	7.06	1778	92.94	*0*.*62*		N/A		N/A	
Primigravida	26	7.81	307	92.19			N/A		N/A	
**Marital Status**										
Married	161	7.47	1994	96.55	*0*.*07*	23	8.42	250	91.58	*0*.*59* ^a^
Other	5	3.45	140	92.53		3	5.00	57	95.00	
**Distance to Healthcare Facility**										
0-5km	54	6.74	747	93.26	*0*.*61*	9	8.11	102	91.89	*0*.*87*
5-10km	61	7.03	807	92.97		10	8.55	107	91.45	
10+km	50	8.06	570	91.94		7	6.73	97	93.27	
**HIV status**										
Negative	136	7.23	1745	92.77	*0*.*24*	21	7.42	262	92.58	*1*.*00*^a^
Positive	2	3.28	59	96.72		0	0.00	8	100	
**Sickle Cell Status**										
AA-normal	99	7.25	1267	92.75	*0*.*67*	14	6.67	196	93.33	*0*.*55* ^a^
AS/AC/SS-carrier	25	6.61	353	93.39		5	9.43	48	90.57	
**Anemia**										
No	72	6.50	1036	93.50	*0*.*19*	12	7.23	154	92.77	*1*.*00*
Yes	52	8.16	585	91.84		7	7.22	90	92.78	

Notes:

* Significance based on Pearson's Chi-square for Fisher's Exact p<0.05 significant

^a^Indicates p-value based on Fishers Exact

**Table 2:** Crude and logistic regression models of the odds of C-Section vs vaginal birth.

	Full Sample				Primigravida			
	Crude	P	Adjusted^a^ (N = 1680)	*P*	Crude	*P*	Adjusted^a^ (N = 257)	*P*
**Mother's Age**								
17–24	*Ref*		*Ref*		*Ref*		*Ref*	
25–34	2.40 (1.40–4.10)*	*<0*.*01*	2.00 (1.03–3.87)*	0.04	1.64 (0.70–3.88)	0.26	0.85 (0.24–3.09)	0.81
35–45	3.74 (2.07–6.73)*	*<0*.*01*	2.69 (1.24–5.82)*	0.01	2.15(0.42–10.87)	0.36	1.61 (0.24–11.02)	0.63
**Education**								
None/Primary	*Ref*		*Ref*		*Ref*		*Ref*	
Secondary	1.19 (0.78–1.84)	0.42	1.26 (0.71–2.23)	0.43	0.92 (0.25–3.43)	0.90	0.57 (0.13–2.58)	0.47
Tertiary	3.35 (2.11–5.32)*	*<0*.*01*	2.85(1.50–5.40)*	*<0*.*01*	2.58 (0.69–9.61)	0.16	0.87 (0.16–4.80)	0.87
**Employment Status**								
Full-time	*Ref*		*Ref*		*Ref*		*Ref*	
Part-time	0.55(0.35–0.86)*	*<0*.*01*	0.55 (0.32–0.96)*	0.03	1.70 (0.52–5.53)	0.38	1.71 (0.43–6.73)	0.45
None	0.77 (0.54–1.10)	0.16	0.80 (0.51–1.24)	0.32	1.51 (0.56–4.06)	0.42	1.43 (0.42–4.83)	0.57
**Residence**								
Urban	*Ref*		*Ref*		*Ref*		*Ref*	
Rural	0.43 (0.31–0.59)*	*<0*.*01*	0.58 (0.38–0.89)*	0.01	0.32 (0.14–0.73)*	*<0*.*01*	0.27 (0.09–0.81)*	0.02
**Malaria Parasitemia**								
High	1.49 (1.03–2.15)*	0.03	1.54 (1.04–2.28)*	0.03	0.61 (0.21–1.75)	0.36	0.68 (0.22–2.09)	0.50
Low	*Ref*		*Ref*		*Ref*		*Ref*	
**Number of People in Household**								
1–2	*Ref*		*Ref*		*Ref*		*Ref*	
3–4	0.96 (0.61–1.49)	0.84	0.94 (0.49–1.80)	0.85	1.62 (0.70–3.72)	0.26	1.39 (0.50–3.89)	0.53
5+	0.67 (0.43–1.06)	0.09	0.57 (0.29–1.16)	0.12	0.27 (0.03–2.10)	0.21	0.37 (0.04–3.13)	0.36
**Gravidity**								
Primigravida	1.11 (0.72–1.73)	0.62	1.04 (0.55–1.96)	0.92	N/A		N/A	
Multigravida	*Ref*		*Ref*		N/A		N/A	
**Marital Status**								
Other	2.26 (0.91–5.60)	0.08	1.12 (0.39–3.23)	0.84	1.75 (0.51–6.02)	0.38	1.47 (0.27–7.96)	0.65
Married	*Ref*		*Ref*		*Ref*		*Ref*	

Notes:

^a^. Models adjusted for other variables in the table

*Indicates significance at p<0.05

### Laboratory measures

Variables assessed by laboratory tests were hemoglobin, malaria parasitemia, human immunodeficiency virus (HIV), and sickle cell disease/trait (SCD). Participants were tested either at baseline—following recruitment into the study—or during their prenatal visits, whereupon records were obtained from the participant’s corresponding hospital.

Hemoglobin was assessed using the standard cyanmethemoglobin method [32]. WHO guidelines for anemia were employed [33], and pregnant woman were classified as anemic if they had a hemoglobin level below 11g/dl.

Peripheral parasitemia levels were assessed using the malaria plus system [34]. Because results indicated that 99% of this sample showed malaria parasitemia, malaria parasitemia was reclassified as low and high based on the malaria plus system with those in the 0 and + group classified as low parasitemia and those in the ++ and +++ groups classified as high parasitemia.

HIV testing was performed using the Rapid Testing Serial Algorithm II [35]. If both tests were positive for HIV, the individual was considered HIV positive; if both tests were negative, the individual was considered HIV negative. When the tests showed conflicting results, they were both repeated and the results were read by another technician, who did not know the results of the first series of tests.

EDTA-treated venous blood samples were used to screen for SCD. To decrease the chances of a false positive or negative of SCD, each sample was tested twice. If incongruent results occurred, the test was rerun.

### Statistical methods

Univariate analyses were based on Pearson's Chi-square test for comparison of proportions for all variables. Fisher's exact tests for contingency tables were used to test for significance in proportions when the expected cell counts were less than 5. Chi-square analyses with p<0.10 were further analyzed using crude and adjusted logistic regression with CS as the main outcome. Having a CS in previous pregnancies is known to predict current CS; therefore, gravida was included in logistic regression models. Because no information was collected specifically regarding previous CS, a sensitivity analysis was performed among those experiencing their first pregnancy. Statistical significance was set at p<0.05. An adjusted trend in the Odds Ratio (OR) was conducted to determine whether there was an increasing trend in the odds of having a CS as a participant’s age and education level increased by using the “tabodds” function in Stata [Stata Corporation, College Station, TX]. Participant’s age was recorded during pregnancy on the baseline survey and was categorized as 17–24, 25–34, and 35–45. Only one women who had a CS had no formal education; therefore, education was categorized as none/primary, secondary and tertiary and above. Age and education were retained as categorical variables for inclusion in multivariable models. Birthweight was collected as part of the parent study; however, because it was self-reported and most newborns were not weighed at birth, birthweight was not deemed reliable. Therefore, birthweight was not included in this analysis. A power analysis was conducted in the parent study [30]; because mode of delivery was not the main outcome of the trial, no additional power analyses were completed before data collection. No difference was observed in mode of delivery between the control and intervention groups (CS intervention group 7.20%, control group 8.54%; mode of delivery chi-square: p = 0.27). Therefore, data was not restricted to only the control group and all analyses treated the sample as a cohort. Data analyses were conducted using Stata version 12.0. The parent study was approved by the Institutional Review Board of the University of Nevada, Reno, and the Nigerian National Health Research Ethics Committee. This secondary data-analysis was appraised by Research Office of the Mel and Enid Zuckerman College of Public Health, and was considered exempt. This research was funded by the Eunice Kennedy Shriver National Institute of Child Health and Human Development (NICHD), the National Institute of Mental Health (NIMH) and the President’s Emergency Plan for AIDS Relief (PEPFAR) under award number R01HD075050 to Echezona Ezeanolue, MD.

## Results

Results from univariate analyses are shown in Table 1; 166 (7.22%) women had CS and 2134 (92.78%) had vaginal deliveries. A woman’s age was statistically associated with having a CS (*p<0*.*01*), with a greater percentage of women aged 35–45 having had a CS (11.22%) than women aged 25–34 (7.05%) or 17–24 (3.27%). Education was statistically associated with having a CS (*p<0*.*01*), with a greater percentage of women with tertiary education having had a CS (15.06%) than those with a secondary education (5.95%) or a primary education or less (5.03%). Employment status was also statistically associated with having a CS (*p = 0*.*03*); women with full-time employment had higher percentages of CS than women who worked part time or who did not indicate they were currently employed (8.72%, 5.01%, and 6.89%, respectively). Area of residence (i.e., rural vs urban), was significantly related to having a CS (*p<0*.*01)* with more women in urban settings having a CS (12.00%) compared to women in rural settings (5.55%). A mother’s baseline malaria parasitemia was significantly associated with having a CS (*p = 0*.*03)*; more women with high malaria parasitemia at baseline had a CS than women who displayed low levels of malaria parasitemia (8.80% vs 6.08%, respectively). No significant relationship was observed between CS and number of people in the household, gravidity, distance to nearest healthcare facility, marital status, HIV, sickle cell disease, or anemia. When analyses were restricted to women who did not have a prior pregnancy (n = 333), 7.81% (n = 26) had CS and 92.19% (n = 307) had vaginal deliveries (Table 1). Among these primigravida women, living in an urban environment was significantly related to having a CS (*p<0*.*01*) with 14.29% of urban women having a CS compared to only 5.11% of rural women. No other participant characteristics were significant predictors of having a CS among primigravida women.

Table 2 presents the crude and adjusted odds ratios (95% CIs) for having had a CS or a vaginal birth by participant characteristics. The adjusted models showed that, compared to women aged 17–24, the odds of having a CS were higher when the mother was aged 25–34 years (adjusted OR (aOR): 2.00; 95% CI: 1.03–3.87) and when the mother was aged 35–45 years (aOR: 2.69; 95% CI: 1.24–5.82; *p-trend <0*.*01*). Compared to those with a primary school education or less, the odds of having a CS were higher if the mother had at least a tertiary education (aOR: 2.85; CI: 1.50–5.40), but not if she had a secondary education (aOR: 1.26; CI: 0.71–2.23; *p-trend <0*.*01*). The odds of having a CS were significantly lower if participants were employed part-time compared to full-time (aOR: 0.55; CI: 0.32–0.96). Compared to women who lived in an urban setting, those who lived in a rural setting had a significant reduction in the odds of having a CS (aOR: 0.58; CI: 0.38–0.89). Significantly higher odds of having a CS were seen among those with high peripheral malaria parasitemia compared to those with low parasitemia (aOR: 1.54; CI: 1.04–2.28). After adjustment for confounders, no relationship was found between CS and number of people in the household, gravidity, marital status and not being employed. Among primigravida women, adjusted logistic regression models showed a significant relationship between woman living in an urban vs rural environment and having a CS (aOR: 0.27; CI: 0.09–0.81). No other significant relationships were found among primigravida women between having a CS and a woman’s baseline characteristics.

## Discussion

Women in SSA continually struggle to obtain adequate obstetric care. Increased access to emergency obstetric care, such as CS, decreases maternal and infant morbidity and mortality [18, 19]. Nigeria has one of the fastest growing populations in the world, making it a key location to study access to healthcare in pregnancy and delivery. Overall results indicate that 7.22% of women in Enugu, southeast Nigeria had a CS while 92.78% had a vaginal delivery. Percentages of CS increased as maternal age and/or education increased. Compared to women who had full-time employment, women who worked part-time had 45% lower odds of having a CS after adjusting for potential confounders. Likewise, significantly lower odds of having a CS were observed among women who live in a rural setting compared to those who reside in an urban setting in both the full sample and among primigravida women. After adjustment for confounding, this study demonstrated 54% higher odds of having a CS if participants had high peripheral malaria parasitemia compared to those with lower peripheral malaria parasitemia.

The present work demonstrated higher percentages of CS in older women and those with more education. In SSA, education has been shown to be a strong predictor of using professionally-assisted delivery services [11, 36]. Older and more educated women in SSA are thought to be more confident and influential in their household decision-making, including the use of healthcare services [11, 37]. Likewise, women with more education and/or women who are employed often have greater control over family resources and play a larger part in reproductive decision-making [17, 37, 38]. These variables may be a proxy of a woman’s ability to access healthcare, thereby increasing her chances of having had a CS.

Although the results did not demonstrate an association between having had a CS and distance to nearest health facility, there was a statistically significant relationship between having had a CS and living in rural vs urban environments. Distance has consistently been an important barrier to seeking healthcare in rural settings [10, 12, 39]. It is possible that in this self-report study, area of residence (i.e., rural vs urban) was an indirect assessment of the ease of reaching a healthcare facility for childbirth. Women living in rural parts of Enugu State, Nigeria—like women in other rural areas—may have had increased difficulty accessing healthcare facilities that can perform a CS because of limited transportation options, poor road conditions, and poverty [10, 12, 17]. Women in rural areas also refrain from using healthcare facilities during pregnancy because of financial constants, cultural norms that discourage delivery in a hospital setting, or gender inequalities in household decision-making [10, 12, 17]. Therefore, it remains unclear if distance to the nearest healthcare facility was a measure of difficulty accessing a healthcare facility or willingness to use a healthcare facility and it does not indicate if the nearest facility had CS access.

To the authors’ knowledge, this study is the first epidemiological investigation to report that high malaria parasitemia is associated with higher odds of CS. The literature and current guidelines are based on case studies [40–42]. It is unknown if a biological pathway exists underlying this relationship, or if women with higher malaria parasitemia lack adequate health care overall, which inherently makes their pregnancies higher risk. The relationship between malaria parasitemia and CS warrants further attention.

In SSA, some evidence has suggested that women with SCD are more likely to have a CS [43, 44]. However, it is difficult to determine whether those with SCD receive any benefits from having a CS, because both SCD and CS are related to high risk of adverse maternal and neonatal outcomes in SSA [44–46]. It has been established that malaria and SCD are associated with anemia during pregnancy [47–50]. There has been much debate in the literature on whether anemia is related to an increase in maternal morbidity and mortality in the context of developing countries [51]. Having a CS would complicate this relationship and also warrants further attention.

Our study is consistent with a meta-analysis that found HIV-infected women were no more likely to have a CS than those uninfected [52]. Evidence from resource unconstrained areas suggests that having a CS is beneficial if a woman’s HIV-RNA level is above 1000 copies/ml near delivery [53]. Because women in resource-constrained areas are often unaware of their viral load before delivery, having a CS could be beneficial for HIV-infected women [54]. However, in resource-constrained areas, CS are often unavailable and unsafe; therefore, the WHO guidelines do not currently recommend that HIV positive women in resource constrained regions have an elective CS [55]. Instead, the WHO recommends that HIV positive women take 3 or more antiretroviral medications in order to decrease mother to child transmission of HIV [56]. The WHO also recommends that infants receive antiretrovirals during the post-natal period if their mother is HIV positive [56].

### Implications for practice and policy

When medically necessary, CS are frequently lifesaving procedures. However, risks associated with CS are often highest within African countries as medical personnel may lack the training to perform a safe CS and lack proper equipment and supplies [6, 12]. In general, maternal and newborn deaths decrease as a population’s CS rates approach 10% [7]. Although the CS rate of 7.22% in our sample fell below this level, the WHO has estimated—based on rates of fistula—that in 15.5% of pregnancies in Nigeria, a CS is medically necessary [1]. This is double the rate found in our sample. Additionally, because of contact with study personnel it is possible the true population estimate may be lower. This indicates that unlike in other parts of the world where discussion centers on overutilization of CS [1], it is likely that in this area of Nigeria, there is an overall underutilization of CS, particularly in rural settings where only 5.55% of all births were delivered via CS.

Although many countries in SSA have healthcare facilities that can perform CS, the quality of care within these clinics is neither consistent nor reliable [57]. It is estimated that less than 1% of individuals in Western SSA have access to surgical care that is safe, affordable and can be performed in a timely manner [58]. Countries in SSA suffer from an overall shortage of facilities equipped to perform such specialized treatment; additionally, many countries in SSA suffer from a lack of skilled workers capable of performing specialized medicine [10]. Because previous research in Nigeria showed that only 1 in 21 health facilities were equipped to perform CS [26], it is likely that even if access to health clinics were increased, most clinics would not be equipped to perform CS safely. Increased access to trained healthcare personnel who can identify and perform life-saving obstetric procedures is needed, especially in rural areas of Nigeria.

### Strengths, weaknesses and future research

This study is unique to the literature on CS in SSA given that it explored the relationship between CS and socio/demographic variables as well as different disease statuses. This allowed us to capture a holistic understanding of risk factors associated with CS in Nigeria. However, the study was not without limitations. In the overall sample, the reasons a woman had a CS were not established, nor was prior use of CS. Because our findings are derived from analysis of secondary of data, indications for having a CS could not be ascertained. The hypothesis that most CS in the study setting were for emergency complications deserves further investigation. It is possible that irrespective of obstetric indication, women with higher education and those in full time jobs could have opted for an elective CS. It is also important to think about factors such as pain management during vaginal delivery and how this affects use of CS particularly among those that have access to this service. Also, the role of cultural preference for vaginal delivery even when a CS is medically indicated, deserves elucidation. An attempt was made to control for prior CS by using the number of people in the household as well as gravidity status as proxies for prior CS; however, it is unknown if this control method was adequate. A sensitivity analysis was also performed using only primigravida women; but the number of women (N = 26) that had a CS for their first pregnancy was small. Therefore, significant relationships between CS and other variables may not have been present in this study because of lack of power. In the parent study, only the intervention group was provided onsite laboratory disease testing. Therefore, participants in the control group were more likely to have missing disease data. It is unknown if this changed the relationship between disease status and mode of delivery. Also, no follow-up occurred when women did not attend post-natal interviews. Thus, it was not possible to determine the rate of maternal mortality. Because all women in this study were self-identified as pregnant and no gestational age estimates were collected, the time between baseline survey and follow-up exit interviews when mode of delivery was ascertained varied and it is unknown how this effected the results. Finally, it is unknown whether women in rural settings truly had difficulty accessing a healthcare facility; perhaps more women in urban settings elected to have a CS. However, given the WHO estimates that 15.5% of pregnancies in Nigeria need a CS, it is likely that women in both rural and urban areas need better access to hospitals with supplies and trained doctors to perform a CS. Answering these questions is essential to understanding the barriers that women face when seeking adequate perinatal care in Nigeria.

In conclusion, rates of CS remain substantially lower in Nigeria than suggested by the WHO Working Group on Caesarean Section [7]. Findings from this study reveal that contrary to the increasing trend in use of CS in low- and middle-income countries and globally, the rate of CS among women in the study setting was low. Further research is needed to ascertain the obstetric conditions under which women in this region receive CS.
